# Gut Microbiota: Association with Fiber Intake, Ultra-Processed Food Consumption, Sex, Body Mass Index, and Socioeconomic Status in Medical Students

**DOI:** 10.3390/nu16234241

**Published:** 2024-12-09

**Authors:** Laura Moreno-Altamirano, Karina Robles-Rivera, Hugo G. Castelán-Sánchez, Felipe Vaca-Paniagua, María del Carmen Iñarritu Pérez, Sandra Elvia Hernández-Valencia, Carlos Cruz-Casarrubias, Juan José García-García, Miguel Ruíz de la Cruz, Héctor Martínez-Gregorio, Clara Estela Díaz Velásquez, Guadalupe Soto-Estrada, Armando Navarro-Ocaña, Santiago Carrillo-Medina

**Affiliations:** 1 Public Health Department, Faculty of Medicine, National Autonomous University of Mexico (UNAM), Avenida Universidad 3000, Copilco, Coyoacán, Mexico City 04510, Mexico; lamorealmx@yahoo.com.mx (L.M.-A.); mcipq@yahoo.com.mx (M.d.C.I.P.); jgarcia@facmed.unam.mx (J.J.G.-G.); gumikar@gmail.com (G.S.-E.); arnava@unam.mx (A.N.-O.); 2 Research Department, Secretariat of Clinical Education, Medical Internship and Social Service, Faculty of Medicine, National Autonomous University of Mexico (UNAM), Avenida Universidad 3000, Copilco Universidad, Coyoacán, Mexico City 04510, Mexico; 3 Department of Pathology and Laboratory Medicine, Western University, Dental Sciences Building, Rm. 4044, London, Ontario N6A 5C1, Canada; hcastelans@gmail.com; 4 Laboratorio Nacional en Salud, Diagnóstico Molecular y Efecto Ambiental en Enfermedades Crónico-Degenerativas, Facultad de Estudios Superiores Iztacala, National Autonomous University of Mexico (UNAM), Tlalnepantla 54090, Mexico; felipe.vaca@iztacala.unam.mx (F.V.-P.); hector.martinez@iztacala.unam.mx (H.M.-G.); cdiazvelasquez@aol.com (C.E.D.V.); 5 Unidad de Investigación en Biomedicina, Facultad de Estudios Superiores Iztacala, National Autonomous University of Mexico (UNAM), Tlalnepantla 54090, Mexico; miguelruiz3541@gmail.com; 6 National Institute of Rehabilitation Luis Guillermo Ibarra Ibarra, Calzada Mexico-Xochimilco 289, Arenal de Guadalupe, Tlalpan, Mexico City 14389, Mexico; sandrahv710@gmail.com; 7 Center for Nutrition and Health Research, Mexican National Institute of Public Health, Fray Pedro de Gante 12, Belisario Domínguez Sección 16, Tlalpan, Mexico City 14080, Mexico; casarrubiasnt@gmail.com; 8 Centro de Investigación Trials in Medicine S.C., Avenida Álvaro Obregón 121 Floor 15 Suite 1504, Cuauhtemoc, Mexico City 06700, Mexico; santiagocarrillomedina31@gmail.com

**Keywords:** diet, ultra-processed food, fiber intake, gut microbiota, overweight, obesity, socioeconomic status, medical student

## Abstract

The gut microbiota plays a vital role in various physical and physiological processes, including immune system regulation, neurotransmitter production, inflammatory response modulation, and the inhibition of pathogenic organisms. An imbalance in the microbial community, known as dysbiosis, has been associated with numerous health issues. Biological influences, health behaviors, socioeconomic determinants, and nutritional status can disrupt this balance. Objective: To evaluate the differences in the gut microbiota composition in medical students according to fiber intake, ultra-processed food (UPF) consumption, sex, body mass index, and socioeconomic status. Methods: A cross-sectional study was conducted with 91 medical students, and 82 fecal samples were analyzed. Sociodemographic and dietary data were collected via questionnaires, UPF consumption was assessed using the NOVA classification, and trained nutritionists performed anthropometry. DNA extraction and 16S rRNA sequencing were performed for the microbial analysis. Bioinformatics and statistical tests included the Dunn and Kruskal–Wallis tests, a PCoA analysis, PERMANOVA, ANOVA, Spearman’s rank correlation, and alpha and beta diversity metrics. Results: Dietary fiber intake strongly influences gut microbiota composition. Lower fiber intake was associated with a higher prevalence of *Parabacteroides* and *Muribaculaceae*. *Prevotella* was more prevalent in individuals with lower UPF intake, while *Phascolarctobacterium* was prevalent in those with higher UPF consumption. Significant differences were associated with sex and UPF consumption but not BMI or SES. Women consumed more UPF, which correlated with distinct gut microbiota profiles. Conclusions: This study highlights the significant impact of diet, particularly fiber intake and UPF, on gut microbiota composition, emphasizing the importance of dietary habits in maintaining gut health.

## 1. Introduction

The microbiota, a functional and essential human body component, consists of trillions of microorganisms from thousands of different species—including bacteria, viruses, fungi, and parasites—residing in the oropharynx, respiratory and gastrointestinal tracts, genitalia, and skin. The gut microbiota predominantly consists of the phyla *Firmicutes*, *Bacteroidetes*, *Actinobacteria*, *Verrucomicrobia*, *Proteobacteria*, and *Euryarchaeota*. *Firmicutes* represent the most abundant phylum in adults considered clinically healthy, followed by *Bacteroidetes* and *Actinobacteria* [1].

The gut microbiota plays a crucial role in multiple physiological processes, encompassing neural and endocrine signaling pathways; metabolic functions such as nutrient absorption, energy harvesting, and vitamin synthesis; and immune system regulation, neurotransmitter production, and inflammatory response modulation [2]. In addition, gut bacteria maintain the mucosal barrier and inhibit the growth of pathogenic organisms, offering protection against infections [1,3,4].

A balanced gut microbiota, or symbiosis, is crucial for maintaining overall health, while imbalances, known as dysbiosis, have been linked to various chronic diseases. However, the impact of health behaviors—such as diet and stress—on this complex system, particularly in high-stress populations like medical students, remains underexplored [3]. Multiple factors influence gut microbiota composition, including biological traits like sex, as well as health-related behaviors and social determinants such as dietary choices, physical activity, sleep, nutritional status, medication use, environmental exposures, air pollution, socioeconomic status (SES), and psychosocial stressors [1,2,4].

Differences in gut microbiota composition have been associated with dietary choices, particularly the consumption of fiber-rich versus low-fiber foods and fresh versus processed foods. Compared with frozen, canned, or ultra-processed foods (UPFs), fresh vegetables and fruits are associated with a more favorable gut microbiota profile.

Previous research underscores the vital link between dietary fiber intake and gut microbiota composition. It shows that higher fiber consumption enhances beneficial bacteria like *Bifidobacteria* and *Faecalibacterium*, increasing the production of short-chain fatty acids (SCFAs) essential for gut health [5]. Dietary fiber acts as a prebiotic, promoting fermentation that generates SCFAs, which support gut barrier integrity [5,6]. A diverse fiber-rich diet fosters a healthier gut microbiome, while low fiber intake is associated with decreased microbial diversity and higher levels of pathogenic bacteria [7]. However, little research has been performed on how these dietary patterns manifest in populations like medical students, who are exposed to stress and poor dietary choices.

UPFs, classified by the NOVA system, are highly processed formulations high in sugars, fats, and sodium but lacking fiber and live bacteria [3,4,8]. Metagenomic studies show that UPF consumption is linked to dysbiosis, marked by reduced microbial diversity, an imbalance between beneficial and harmful bacteria, increased intestinal permeability, and inflammation [9]. These dysbiotic changes are associated with a higher risk of chronic diseases such as obesity, cardiovascular disease, gastrointestinal disorders, type 2 diabetes, and metabolic syndrome [4,8,10,11,12].

Emerging evidence indicates that the gut microbiota of individuals with obesity differs significantly from that of those with a normal weight, highlighting a distinct microbial composition associated with a higher body mass index (BMI). This often includes reduced bacterial diversity and an altered *Bacteroidetes*-to-*Firmicutes* ratio [13].

On the other hand, it has recently been stated that SES plays a vital role in the microbiota composition throughout life, one that is even more substantial than the genetic component. A lower SES has been associated with specific bacterial taxa that may influence susceptibility to chronic diseases [2].

Medical students experience unique stressors [14] and irregular schedules due to their training demands, which can negatively impact their diet, health behaviors, and gut microbiota composition. Higher education institutions can be very stressful for students, and it is especially pronounced in medical careers due to their intense workload. Medical students often have limited time to absorb much information and face considerable pressure from their instructors. This high stress level has been linked to changes in the microbiota composition, which plays a crucial role in individuals’ mental and psychological well-being [15].

This study aimed to evaluate the differences in the gut microbiota composition in Mexican medical students according to fiber intake, UPF consumption, sex, BMI, and SES.

By examining the effects of UPF consumption, fiber intake, and other key factors, this research provides valuable insights into how dietary habits and socioeconomic conditions shape gut health. Understanding these relationships is not only crucial for improving the health and well-being of medical students, but it may also inform dietary and behavioral interventions that promote better health outcomes in other high-stress populations.

## 2. Materials and Methods

### 2.1. Study Design and Characteristics of the Research Project

This study employed an analytical cross-sectional design involving 91 medical students. This group was selected as the study population due to their unique vulnerability to academic and life stressors, which are known to influence gut health. Additionally, this population was accessible and practical to study, given the project’s time and resource constraints. Their participation also offered them valuable early exposure to health research and educational experience, which they could apply in their future medical practice and personal lives.

Participants were recruited through direct personal contact, ensuring a high response rate and engagement with the study. Eligibility criteria included being a medical student aged 18 years or older and abstaining from antibiotics or pharmaceutical probiotics for at least 14 days before participation, as these could influence gut microbiota composition. Detailed information about the study’s objectives and procedures was provided, and informed consent was obtained from all participants.

On the first day, participants completed a self-administered questionnaire to collect data on sociodemographic information, SES, and dietary intake, including UPF consumption and fiber intake. Participants were then given clear instructions on collecting and transporting stool samples for microbiota analysis. On the second day, anthropometric measurements (e.g., weight, height, BMI) were taken to assess their physical characteristics. Stool samples were subsequently collected and preserved using standardized methods to ensure the integrity of the samples for microbiota analysis.

### 2.2. Assessment of Ultra-Processed Food Consumption and Fiber Intake

Food intake was assessed using a semi-quantitative food frequency questionnaire. It included 152 food and beverage items and questions about portions consumed seven days before its application. The food frequency questionnaire (FFQ) was based on the instrument developed for the National Health and Nutrition Survey (ENSANUT), a nationally representative survey essential for assessing health and nutrition trends and informing public health policies and interventions in Mexico [16]. UPF was classified according to the NOVA food classification system [17], considering their nature and the degree of processing. UPFs included industrialized drinks (soft drinks, flavored drinks, nectars, and drinks with sweeteners), fried foods, sausages, boxed cereal, and fast food (pizzas, hamburgers, and hot dogs) [18] (Table A1). Although the NOVA framework provides useful distinctions, classifying certain foods is not always straightforward. Four independent researchers reviewed product labels to ensure consistency, resolving discrepancies by consensus. Foods were classified as ultra-processed only if they contained multiple additives beyond simple preservatives or salt. To enhance transparency, we documented decisions for borderline cases. While the degree of processing does not always align directly with nutritional quality, NOVA remains helpful for exploring how additives in UPFs relate to outcomes such as gut microbiota changes. However, these nuances highlight the importance of cautious interpretation, particularly for foods in gray areas between minimally processed and UPF categories. The NOVA criteria are subject to ongoing debate and revisions, but they have helped clarify the distinction between natural or minimally processed foods and UPFs. While some discussions, particularly those related to nutritional composition (e.g., macronutrient content), challenge the NOVA system, its framework is still helpful in understanding how certain elements of UPFs—especially food additives—are linked to outcomes such as changes in gut microbiota composition. Previous research, mainly observational studies, has highlighted this connection, attributing the effects of high UPF consumption on the gut microbiota to factors like additives, excessive levels of certain nutrients, and low fiber content [9]. These findings underscore the importance of considering not only macronutrients but also the broader context of food processing and additives when assessing the health impacts of diet.

Procedures from previously published works using this questionnaire were followed [16]. First, the daily consumption amount for each food was estimated by multiplying the number of days by the number of times per day each item was consumed over seven days. Subsequently, this was multiplied by the number of servings per day (servings/day), and the amount consumed (g/day) was estimated based on the number of servings per day. The energy intake and fiber per item (kcal/day/food and fiber/day/food) were estimated using different sources to obtain food composition values, including energy, nutrients, and fiber [18,19]. The high-fiber food groups included fruits, vegetables, legumes, cereals, tubers, and healthy fats; and foods commonly consumed in the study region, including Mexican staples such as corn tortillas and nopales. At the same time, low-fiber foods were considered UPFs. Both nutritional components were assessed according to the amount of each item consumed. Energy intake per person per day (kcal/day) was calculated by summing the kcal/day/food.

For dietary data processing, consumption in grams per day (g/day) for each food item was analyzed, with extreme intake values being identified as those falling within four standard deviations, with two on either side of the mean. Outliers were replaced with random values within each food group’s 90th to 95th percentile distribution range. After correcting the intake vectors, the same method was applied to energy intake (kcal/day). The energy contribution of UPFs was then estimated by calculating the percentage of total energy intake each participant derived from these items.

Moreover, the calculated energy contribution (%) of UPFs was classified into tertiles and analyzed according to the national consumption reported of 35.5% [19], obtaining two categories: above (>35.5%) and below (<35.5%) the national consumption average.

According to the recommendation of the World Health Organization (WHO) regarding a fiber intake of 25 g per day [20], the students were classified into two categories: Those who accomplished the recommendation (≥25 g per day) and those who did not achieve it (<25 g per day).

### 2.3. Anthropometric Measurements

Anthropometric measurements were collected by trained nutritionists utilizing standard validated methods, including a calibrated stadiometer and a Tanita scale for measuring height and weight. BMI was calculated and subsequently classified according to the WHO standards for adults: normal weight (<25.0 kg/m^2^), overweight (25.0–29.9 kg/m^2^), and obesity (≥30 kg/m^2^). For analytical purposes, participants were grouped into normal weight and overweight + obesity [21].

### 2.4. Socioeconomic Status (AMAI Rule)

Socioeconomic status was assessed using the AMAI rule, a scoring system based on a statistical model designed to categorize Mexican households into seven levels (A/B, C+, C, C−, D+, D, and E) based on their ability to meet basic needs [22]. The score incorporates data on the educational level of the household head, the number of rooms and bathrooms, car ownership, employment status, and Internet access. However, given the homogeneity of the characteristics of the participants and the necessity for Internet access during the COVID-19 pandemic to continue their medical education, the SES score was adjusted to exclude Internet access as a variable. Percentile scores for each item were then calculated, and the seven SES levels were consolidated into three categories: low (D and E), medium (C, C−, and D+), and high (A/B and C+).

### 2.5. Fecal Sample Self-Collection and Preservation, DNA Extraction, Bacterial 16S rRNA PCR Sequencing, and Bioinformatic Analysis

Participants self-collected fecal samples, with approximately ten grams of stool from the first bowel movement of the day preserved in a container with transport and preservation buffer (DNA/RNA Shield, Zymo Research, R1101-1, Irvine, CA, USA) and stored at 4 °C until DNA extraction. Of the 91 collected stool samples, 82 were viable for analysis, as 9 samples were insufficient for DNA extraction.

DNA extraction was performed with the ZymoBIOMICS DNA miniprep kit (D4304), following the manufacturer’s instructions. Fecal sample DNA was then sequenced via Zymo Research’s ZymoBIOMICS service, targeting the bacterial 16S rRNA gene with the Quick-16S™ NGS library preparation kit.

Region V3–V4 of the 16S rRNA gene was amplified with PCR with the primer-Illumina adaptors, forward TCGTCGGCAGCGTCAGATGTGTATAAGAGACAGCCTACGGGNGGCWGCAG and reverse GTCTCGTGGGCTCGGAGATGTGTATAAGAGACAGGACTACHVGGGTATCTAATCC. The PCR was performed in 50 μL reaction mixtures at a final concentration of 0.02 μM. PCR was performed in 50 uL reaction mixtures at a final concentration of 0.2 uM of primers, GoTaq Green Master Mix 1x, and 50 ng of DNA.The amplifications were performed in a T100 Thermal Cycler (Bio-Rad, Hercules, CA, USA) with the following program: initial denaturation at 95 °C for 3 min, followed by 35 cycles of denaturation at 95 °C for 30 s, primer annealing at 55 °C for 30 s and extension at 72 °C for 30 s, and final elongation at 72 °C for 5 min. PCR products were verified by electrophoresis and purified using AgenCourt AMPure XP magnetic beads (Beckman Coulter, Brea, CA, USA) according to the Illumina 16S Metagenomic Sequencing Library Preparation protocol. Barcodes were then added to the amplicons for sequencing. The samples were normalized by molar concentration, pooled, and sequenced for 2 × 250 cycles in a MiSeq Illumina platform at Laboratorio Nacional en Salud, FES Iztacala, UNAM.

The overall quality of sequencing was evaluated with MultiQC [23]. The paired-end reads were assembled with PEAR [24] and imported into the Quantitative Insights into Microbial Ecology (QIIME2; v.2022.2) software package [25]. Then, the data were denoised with DADA2 to read filtering, dereplication, and chimera removal. Taxonomic classification was performed with a 16S rRNA gene database from Silva 138 classifier [26] (silva-138-99-nb-classifier.qza).

### 2.6. Statistical Analysis

Descriptive statistics were presented using the median (p50) and interquartile range (IQR) for quantitative variables, while absolute frequencies and percentages were used for qualitative variables. Differences in medians were evaluated using the two-sample Wilcoxon rank-sum (Mann–Whitney) test, and proportions were compared using the immediate proportions test, Stata version 14.2 (StataCorp, College Station, TX, USA). A *p*-value of <0.05 was considered statistically significant.

The microbial composition of fecal samples was analyzed and compared based on UPF consumption, sex, BMI categories (normal weight vs. overweight/obesity), and SES. To assess the effects of UPF consumption on gut microbiota, participants were divided into two groups (as described in the Section 2), and UPF consumption was divided into tertiles for analysis. The Dunn test, adjusted by the Bonferroni correction method, was performed at the genus level for each taxon to assess differences in UPF consumption between tertiles. Principal coordinate analysis (PCoA) was used to assess the relative abundance of bacterial genera in the gut microbiota based on UPF consumption.

Alpha diversity metrics, including observed species (amplicon sequence variants, ASVs), the Shannon index, and the Simpson index, were analyzed for low, medium, and high SES levels. ASVs were visualized using boxplots for the Shannon and Simpson indices. Beta diversity was assessed using Aitchison distances calculated from centered, log-transformed relative abundances at the genus level. Differences in beta diversity between BMI categories, SES, gender, and UPF consumption were analyzed using PERMANOVA (*p*-value = 0.001; 999 permutations), following the approach described by Aitchison et al. [27], and ANOVA.

The Dunn post hoc test was used following a Kruskal–Wallis analysis to identify bacterial taxa significantly associated with UPFs and dietary fiber intake. The ANOVA test was used to identify statistically significant differences between the means of the tertiles.

The analysis included all taxa in relative frequency, with frequency data converted to a numerical format before applying Dunn’s test. Taxa with *p*-values ≤ 0.05 were considered significant and provided insight into the effects of UPF and dietary fiber intake on gut microbiota composition.

Spearman’s rank correlation was used to examine the association between bacterial genus abundance and UPF and dietary fiber consumption. This non-parametric method was chosen because it can assess monotonic relationships without the need for normality or linearity assumptions. The analysis was performed using the cormat function in R, which produces a correlation matrix with *p*-values to determine statistical significance.

## 3. Results

### 3.1. Description of the Study Population

A total of 91 medical students with an average age of 20 years were studied, 71.4% of whom were women. The median BMI was 24.4 kg/m^2^ in females and 23.7 kg/m^2^ in males. Overweight status was observed in 33.0% of women and 35.9% of men, and obesity was observed in 7.8% of women and 4.2% of men. Based on the AMAI rule, the SES was high at 13.2%, medium at 61.5%, and low at 25.3% of the participants. Overall, 46.1% of the sample reported consuming UPFs above the national average, with a significantly higher proportion among women (55.4%) compared with men (23.1%) (*p* = 0.005). Conversely, 53.9% of participants reported consuming UPFs below the national average, with 76.9% of men falling into this category compared with 44.6% of women (*p* = 0.005). Regarding fiber intake, 60.4% of participants reported consuming less than 25 g per day, with a slightly higher proportion among women (63.1%) than men (53.9%). In contrast, 39.6% of the sample consumed equal to or more than 25 g of fiber daily, with 46.1% of men and 36.9% of women meeting this recommendation. However, no statistically significant differences in fiber intake between sexes were observed (*p* = 0.417) (Table 1). The average daily fiber intake was 24.8 g (SD 9.9 g). When categorized by tertiles, the mean fiber intake was 14.26 g (SD 3.86 g) in the first tertile, 24.01 g (SD 2.87 g) in the second tertile, and 36.46 g (SD 4.75 g) in the third tertile.

The median energy intake from UPFs in the sample was 863 kilocalories per day (IQR 462.1). Women consumed more than men (895.8 vs. 783.7 kcal). The distribution of energy contribution from UPFs differed between men and women according to tertiles (*p* = 0.004), with a higher percentage of women being in the highest tertile. The proportion of participants in the overweight + obesity group was higher in the highest tertile of UPF consumption than those with normal weight (*p* = 0.202) (Table 2).

### 3.2. Microbiome Composition by BMI and SES

**Figure 1:**
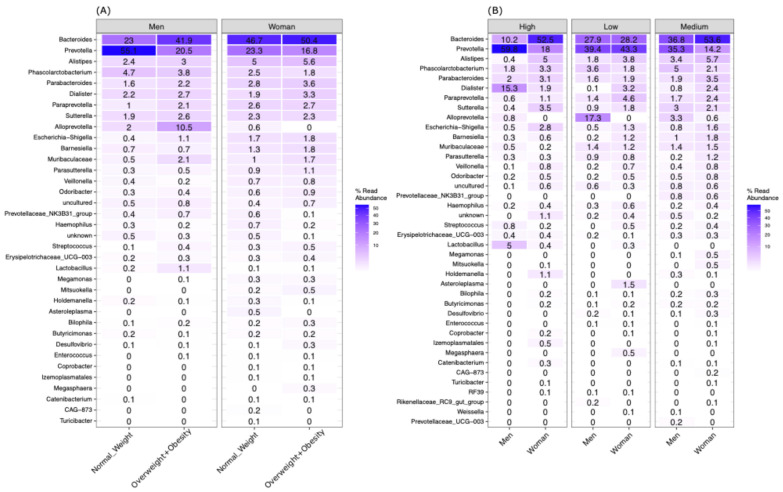
Composition of gut microbiota across different BMI and SES categories by sex. (**A**) Samples grouping according to sex and BMI. (**B**) Samples grouped according to SES and sex.

The microbiota composition across different BMI categories revealed that *Bacteroides* and *Prevotella* were the dominant genera. A detailed analysis showed sex-specific differences: in men, *Bacteroides* (41.9%) was more prevalent among participants with overweight and obesity, while *Prevotella* (55.1%) was more common among men with normal weight. Interestingly, these distinctions were not observed among women (Figure 1A).

Regarding SES, Prevotella (59%) and Dialister (15.3%) were more prevalent in men with high SES. Conversely, Bacteroidetes (27–33%) and Prevotella (35–38%) were the more common in men with medium and low SES, whereas Alloprevotella (17.3%) was more common in men with low SES. In women, the microbiome composition by SES showed that Bacteroidetes and Prevotella were the most abundant genera across all SES levels (ranging from 52% to 25%). Although Bacteroidetes was the most abundant genus in all SES groups, it was less prevalent in low SES men and women (27% and 28%, respectively) (Figure 1B).

### 3.3. Diversity Analysis

**Figure 2:**
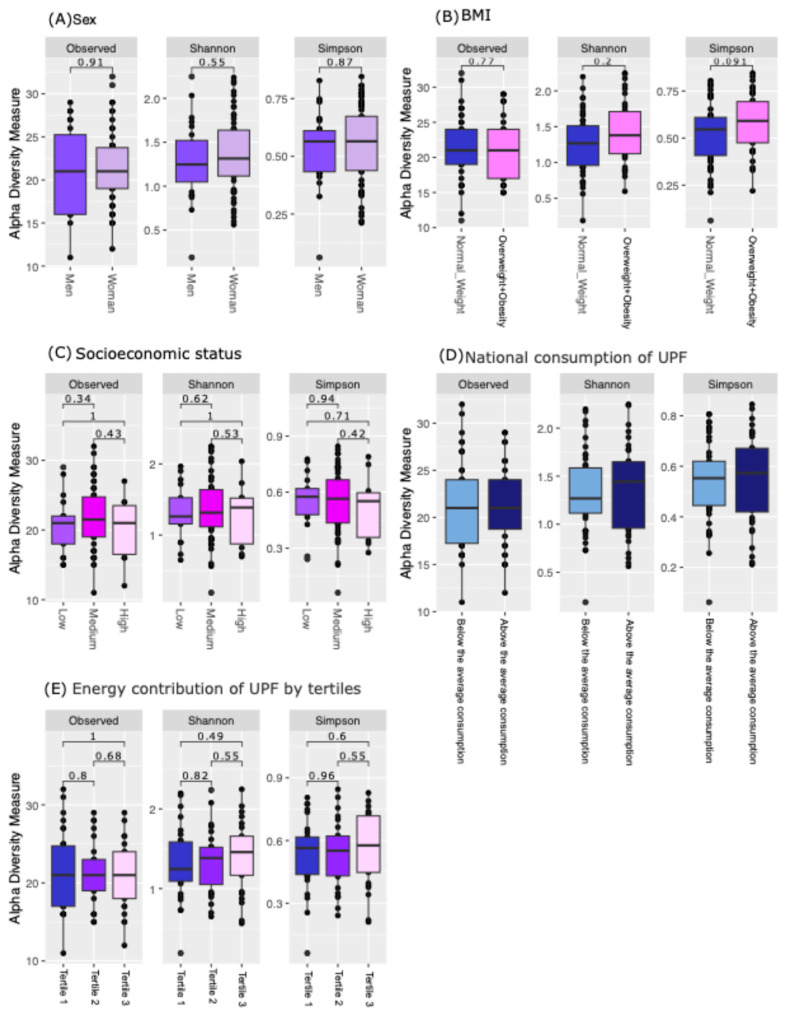
Distribution of alpha diversity metrics (observed ASVs, Shannon, Simpson). (**A**) Sex, (**B**) BMI, (**C**) SES, (**D**) national consumption of UPF, (**E**) energy contribution of UPF by tertiles.

We evaluated the distribution of alpha diversity using the observed ASVs, Shannon index, and Simpson index, aiming to observe differences in microbial diversity between groups. The alpha diversity indices did not show statistically significant variations across the groups evaluated, including sex, BMI, SES, and UPF consumption (Figure 2).

**Figure 3:**
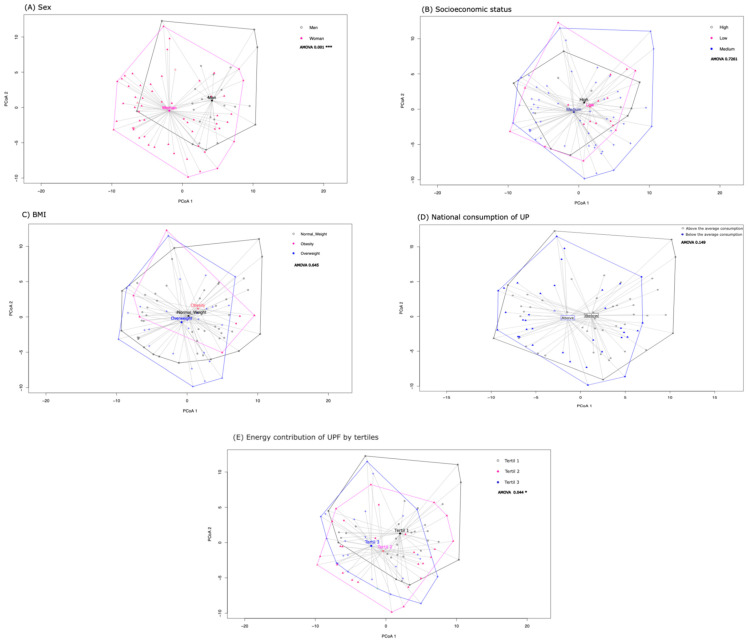
Beta diversity (Atkinson distance). (**A**) Sex, (**B**) SES, (**C**) BMI, (**D**) national consumption of UPF, and (**E**) energy contribution of UPF by tertiles. The points on the graph represent the bacterial communities of each subject, with points closer together indicating more similar bacterial communities.

Beta diversity, an ecological concept describing differentiation in communities’ taxonomic or phylogenetic composition, was analyzed in terms of sex, BMI, SES, consumption of UPF, and UPF energy contribution. Men and women had a highly statistically significant difference in microbiota composition (Figure 3A). Conversely, the ANOVA analysis of BMI categories (normal weight and overweight + obesity) revealed no statistically significant difference, as indicated by a *p*-value of 0.645 (Figure 3B). The microbiome composition across different SESs showed no significant differences (Figure 3C). Similarly, the beta diversity of groups with above- and below-average consumption of UPF revealed no significant differences (Figure 3D). However, a statistically significant difference was observed when dividing groups into three tertiles based on the energy contribution of UPF (*p* = 0.044; Figure 3E). Overall, the ANOVA results suggest significant differences based on sex and UPF consumption, while no significant differences were found for the BMI or SES categories.

Regarding the PCA of the national consumption of UPF, the first principal component, PCoA1, explained 43.6% of the variation in the data, while the second, PCoA2, explained 11.36%. The PCoA diagram indicated notable differences in the gut microbiota of people who consume different amounts of UPF. For example, the genus Prevotella was more abundant in the group that consumed less UPF, while Phascolarctobacterium was more abundant in the group that consumed more.

### 3.4. Ultra-Processed Food and Fiber Intake Modified the Gut Microbiota

**Figure 4:**
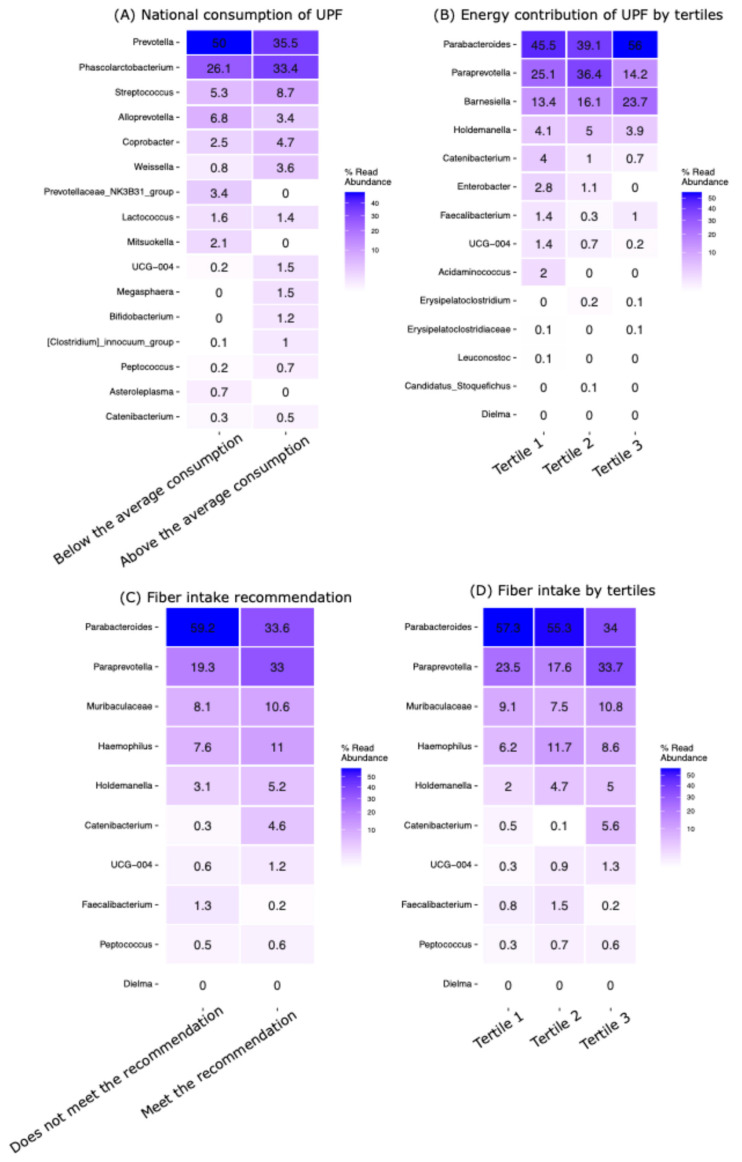
Distribution of genera that were significantly enriched based on UPF consumption and fiber intake (*p*-values ≤ 0.01). (**A**) Genera enriched according to the national consumption of UPF. (**B**) Genera enriched according to the tertiles of UPF consumption. (**C**) Genera enriched in participants according to the meeting of the recommendation of fiber intake. (**D**) Genera enriched in participants with a fiber intake according to tertiles.

The analysis of bacterial taxa revealed significant enrichments (*p* ≤ 0.01) according to UPF consumption and fiber intake (Figure 4). For UPF consumption, Prevotella was significantly enriched in individuals with below-average UPF intake, showing an abundance of 50% compared with 35.5% in those with above-average UPF intake (Figure 4A). Other notable genera include Phascolarctobacterium (26.1% vs. 33.4%) and Streptococcus (5.3% vs. 8.7%).

Further examination of the energy contribution by UPF consumption across tertiles identified Parabacteroides and Paraprevotella as the most abundant genera, with enrichment levels ranging from 14.2% to 56% in the top tertile (Figure 4B). This indicates a substantial prevalence of these taxa in the high-UPF consumption group.

Regarding dietary fiber intake, those who did not meet the daily recommendation exhibited the highest abundance of Parabacteroides (59.2%), compared with 33.6% in individuals meeting the recommendation (Figure 4C). Other abundant genera in this context included Paraprevotella (19.3% vs. 33%) and Muribaculaceae (8.1% vs. 10.6%).

The analysis of fiber intake across tertiles revealed that Parabacteroides remained dominant, with frequencies ranging from 34% to 57.3%, followed by Paraprevotella and Muribaculaceae (Figure 4D).

The results showed that Prevotella, Phascolarctobacterium, and Streptococcus are predominantly enriched in the UPF consumption groups, indicating their potential to thrive in environments influenced by high UPF intake. The enrichment pattern shows that UPF consumers have a higher diversity of enriched genera. In contrast, fiber consumers seem to have fewer but more consistently dominant genera, including a higher dominance of Parabacteroides, Paraprevotella, and Muribaculaceae. Notably, Muribaculaceae appears uniquely associated with fiber intake, suggesting a preference for fiber-rich diets and a potential role in fiber metabolism.

**Figure 5:**
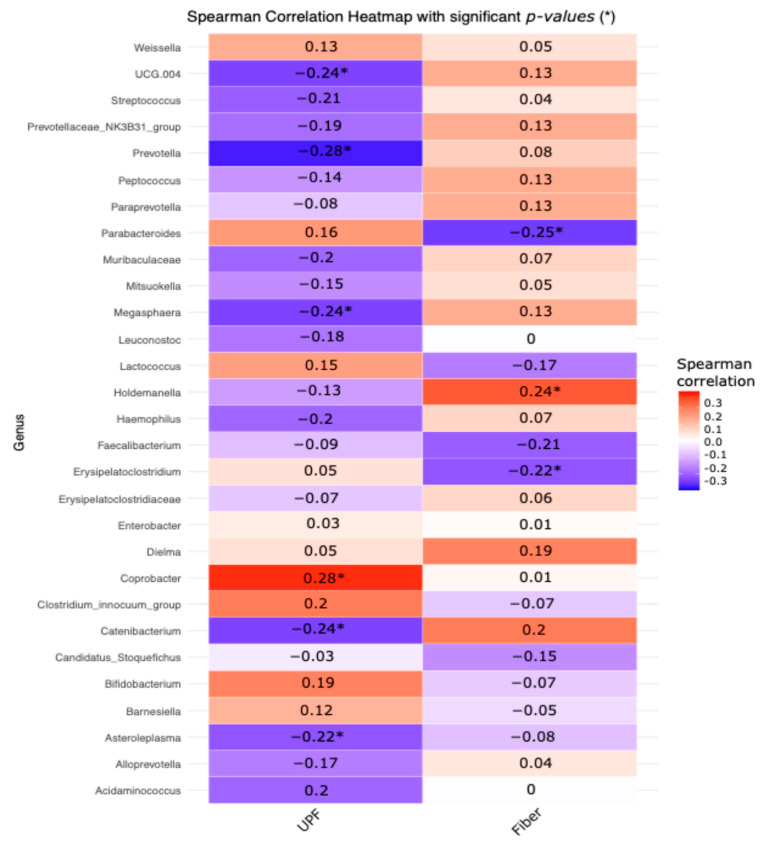
Spearman correlations between bacterial genera and UPF consumption and fiber intake. Asterisks indicate *p*-values < 0.05. A positive Spearman correlation suggests that higher UPF consumption or fiber intake values are associated with a higher relative abundance of the taxa. Negative correlations indicate that higher values of UPF consumption or fiber intake are associated with a lower relative abundance of the taxa.

A Spearman correlation analysis was conducted to evaluate the relationship between bacterial genera and UPF consumption and fiber intake, considering only the genera that were significantly enriched in the previous step (*p*-values < 0.05 in asterisks) (Figure 5). Positive correlations suggest that higher UPF or fiber intake is associated with increased relative abundance of specific taxa, while negative correlations indicate a decrease in abundance. Specifically, Prevotella, Megasphaera, Catenibacterium, and Asteroleplasma showed significant negative correlations with high UPF consumption, whereas Coprobacter displayed a positive correlation. For fiber intake, Holdemanella was positively correlated, indicating increased abundance with higher fiber consumption, while Erysipelatoclostridium and Parabacteroides showed negative correlations, suggesting decreased abundance with higher fiber intake.

## 4. Discussion

Our study highlights significant differences in gut microbiota composition associated with fiber intake and UPF consumption, as well as sex-based variations in a population of medical students. These findings add to the growing body of evidence on the impact of diet on gut health and underscore the importance of dietary habits in shaping the gut microbiome.

Medical students represent a unique population with high levels of mental health problems [28], irregular schedules, and often suboptimal dietary habits [29] due to the demands of their training. These factors make them particularly vulnerable to disruptions in gut microbiota, which can have implications for their physical health and cognitive and emotional well-being. Given the increasing recognition of the gut–brain axis, understanding how dietary choices impact gut microbiota in this population is critical [30]. Our findings suggest that the nutritional habits commonly observed among medical students, such as high UPF consumption and low fiber intake, may contribute to gut dysbiosis, potentially exacerbating stress-related digestive problems and impacting academic performance [31].

### 4.1. Fiber Intake and Gut Microbiota

Conversely, our study found that adequate fiber intake was associated with a gut microbiota profile dominated by beneficial bacteria among students.

Our findings revealed that participants with adequate fiber intake exhibited a notable increase in the abundance of *Holdemanella*, a genus within the *Firmicutes* phylum. *Holdemanella* is linked to several health benefits, such as anti-inflammatory effects, cancer prevention, and improved glucose tolerance through enhanced GLP-1 signaling [32,33]. The increase in *Holdemanella* with higher fiber intake suggests that fiber promotes the growth of beneficial bacteria that thrive on complex carbohydrates, thereby supporting overall gut health [34]. Previous research has similarly shown that increased fiber intake fosters bacterial diversity and beneficial bacteria, including *Bifidobacterium*, *Lactobacillus*, *Akkermansia*, *Faecalibacterium*, *Roseburia*, *Bacteroides*, *Prevotella*, etc., while reducing pathogenic bacteria such as *Enterobacteriaceae* [35].

In contrast, our study found that high fiber intake decreased the abundance of *Erysipelatoclostridium* and *Parabacteroides*. Both genera belong to the *Firmicutes* and *Bacteroidetes* phyla, respectively. The reduction in these genera may reflect a shift in microbial community structure. *Erysipelatoclostridium* is involved in fiber fermentation, so its decrease could indicate a reduced reliance on these fermentation pathways when the fiber is plentiful. Consistent with this, previous studies have shown that higher fiber diets can reduce opportunistic pathogens like *Erysipelatoclostridium*. For instance, a randomized clinical trial found a decrease in *Erysipelatoclostridium* among patients with type 2 diabetes on a high-fiber diet [36]. Additionally, a study of Hispanic or Latino participants over six years found that lower fiber intake was associated with higher levels of *Erysipelatoclostridium* and an increased risk of type 2 diabetes [37].

The effects of fiber on *Parabacteroides* are less clear. Some research indicates that bioactive dietary fibers, such as inulin and various polysaccharides, increase the relative abundance of *Parabacteroides*, particularly *Parabacteroides distasonis* [38]. This discrepancy suggests that the impact of fiber on specific microbial genera may vary based on the type and composition of the fiber consumed and individual differences in gut microbiome profiles. Further research is needed to clarify these differences and understand how dietary fiber influences gut microbial communities.

Our findings highlight the importance of high-fiber diets in fostering a diverse and stable gut microbiota. Undigested dietary fiber is crucial for microbiota health, affecting its composition, abundance, and function. Fiber promotes bacterial growth, fermentation, and the production of short-chain fatty acids, which enhance gut barrier function, modulate immune responses, and contribute to overall gut health. The observed changes in microbial populations with increased fiber intake suggest a more balanced and healthier gut microbiota, with improved metabolic functions and potentially reduced inflammation. This underscores the critical role of dietary fiber in maintaining gut health.

### 4.2. UPF Consumption and Gut Microbiota

Our findings indicate that individuals with a higher consumption of UPFs tend to have gut microbiota characterized by increased levels of genera such as *Parabacteroides*, *Paraprevotella*, *Phascolarctobacterium, Coprobacter*, and *Streptococcus*.

Supporting our findings, Cuevas-Sierra et al. conducted a cross-sectional study with 296 overweight or obese Spanish adults. They found that *Parabacteroides* was significantly more abundant (*p*-value 0.002) in women consuming more than five servings of UPF daily [39]. This genus was previously linked to sugar-sweetened beverages in adolescent rats [40]. Similarly, *Phascolarctobacterium* has been linked with a high-fat, high-sucrose diet in murine models (*q* < 0.02) [41]. In addition, *Coprobacter* (highly similar to *Muribaculum intestinale*) has been associated with diets rich in saturated fat and sugar and has been studied as an indirect measure of adiposity [42]. Another study with 29 healthy native Chinese volunteers showed that those on a high-fat diet had increased levels of *Paraprevotella* (*p*-value 0.035) [43]. Finally, *Streptococcus* has been observed enriched in an animal-based diet rich in cheese and cured meats [44].

Conversely, our study found that higher UPF consumption is associated with decreased abundance of genera such as *Prevotella*, *Megasphaera*, *Catenibacterium*, and *Asteroleplasma*. *Megasphaera* and *Catenibacterium* have been associated with Western diets that include processed foods in subjects with obesity [45]. While there is no published dietary information for *Asteroleplasma*, it has been linked to various inflammatory bowel diseases, including colonic Crohn’s disease, ulcerative colitis, and ileum Crohn’s disease [46,47].

In students with lower UPF consumption, the gut microbiota was dominated by *Prevotella* and *Lactococcus*. *Prevotella* has been extensively studied and is associated with high-fiber, prebiotic, and polyphenol-rich diets, as well as plant-based and Mediterranean diets, which support anti-inflammatory pathways and improve glucose metabolism [35,48]. *Lactococcus*, known for its probiotic benefits, is typically enriched in those consuming long-chain fatty acids, fermented foods, and dairy products, which support a balanced and healthy gut microbiome [35,49].

The observed associations between UPF consumption and altered gut microbiota have significant implications for the health of medical students. Disruptions in gut microbiota due to UPF consumption may worsen stress-related gastrointestinal issues, potentially affecting physical and mental health [34]. Given the growing evidence that gut health impacts cognitive and emotional well-being, promoting healthier eating habits among medical students could improve their academic performance and overall quality of life.

These findings are consistent with the existing literature on the adverse effects of UPF on gut health. UPF is typically low in dietary fiber and high in additives, preservatives, and simple sugars, which do not support beneficial bacteria that thrive in fiber-rich environments. Instead, UPF fosters pathogenic bacteria’s growth, leading to gut dysbiosis [50]. Additionally, additives and preservatives in UPF can impair intestinal barrier function, allowing bacteria and their metabolic products to enter the systemic circulation, thereby exacerbating systemic inflammation and further disrupting gut microbiota composition [51,52]. This cycle of digestive discomfort and metabolic issues underscores the importance of a balanced diet rich in whole foods, which supports a diverse and stable gut microbiome and promotes overall health [53].

### 4.3. Prevalence of Overweight and Obesity

We found a 39.8% prevalence of overweight and obesity in medical students. In Mexico, this prevalence may be influenced by various factors linked to their academic and health behaviors. The rigorous demands of medical training mentioned before may drive students to choose unhealthy, convenience foods as a quick way to manage stress. The resulting lack of time for balanced meal preparation and reliance on UPF contribute to an imbalance between caloric intake and energy expenditure, promoting weight gain and obesity in this group [54].

This prevalence is comparable with the national average of 40.4% for the same age group, as reported by the ENSANUT survey [55], suggesting that medical students are experiencing health challenges similar to those of the population. This similarity highlights broader societal trends affecting health and weight, indicating that medical students are not immune to these general health issues, even with their specialized training. Despite their greater awareness of health and nutrition and access to educational resources, medical students do not show significantly healthier behaviors than the general population. This underscores the need for improved prevention and health promotion strategies targeting the public, health professionals, and medical students. Overall, these findings reflect a significant public health issue in Mexico, where obesity has reached epidemic levels, regardless of the level of education.

### 4.4. BMI, Sex, and Gut Microbiota Composition

Although our study did not find statistically significant differences in BMI-related gut microbiota composition, the observed trends are consistent with previous research. This suggests that while BMI might influence gut microbiota composition, its impact could be mediated by other factors, such as diet and behavior, which were not fully accounted for in our study. Alternatively, the lack of significant findings could be due to the small sample size and homogeneous nature of the population in terms of BMI, with no significant differences being observed between the various BMI strata.

Our analysis found that *Bacteroides* and *Prevotella* were the predominant genera across different BMI categories. Specifically, *Bacteroides* was more prevalent among men with overweight + obesity, while *Prevotella* was more common in men with normal weight. These trends were not observed in women.

Previous studies have associated *Bacteroides* with Western diets high in fats and sugars [56,57]. However, other research has also linked *Bacteroides* to individuals without obesity [58], suggesting that its relationship with BMI might depend on dietary and individual factors. Conversely, *Prevotella* is generally associated with diets rich in fiber and plant-based foods [59].

Our study also revealed sex-specific differences in gut microbiota composition. This finding is consistent with previous research, which has shown that women typically have lower levels of *Bacteroides* than men [60,61]. For example, a study with 230 healthy individuals found that men had higher levels of both *Bacteroides* and *Prevotella* than women [62]. These differences are likely due to a complex hormonal, genetic, and environmental interplay. Hormonal variations, particularly sex hormones like estrogen and testosterone, significantly influence gut microbiota. Estrogen is associated with increased diversity and abundance of beneficial bacteria like *Lactobacillus* and *Bifidobacterium*, whereas testosterone may promote different microbial populations, some of which are linked to inflammation [63,64].

These sex-based differences in gut microbiota composition underscore the need for sex-specific considerations in microbiome research and health interventions, highlighting the importance of personalized gut health and disease management approaches.

### 4.5. Socioeconomic Status and Gut Microbiota Composition

Our study found inconsistent and non-statistically significant results regarding the association between SES and gut microbiota composition. Specifically, *Prevotella* and *Dialister* were more abundant in students from high SES, while *Bacteroidetes, Prevotella*, and *Alloprevotella* were more prevalent in medium and low SES groups. These findings suggest that while SES may influence gut microbiota composition, this effect might be mediated by factors such as diet and behaviors, which were not fully accounted for in our study. Alternatively, the lack of significant findings could be due to the small sample size and the homogeneous nature of the SES distribution in our population, resulting in no statistically significant differences between the SES strata.

SES can influence gut microbiota through its effects on dietary patterns and access to health resources. Individuals from higher SES often have better access to a diverse range of nutritious foods, which can foster a microbiota rich in beneficial genera. In contrast, individuals from lower SES backgrounds may consume diets higher in refined carbohydrates and fats, which can lead to increased levels of *Prevotella copri* and *Catenibacterium sp000437715* [65] as well as a reduction in *Bifidobacterium* [66]. This highlights the importance of considering socioeconomic factors in public health strategies to improve gut health. Addressing the environmental and social factors associated with lower SESs could help mitigate their negative impacts on gut health and overall well-being, emphasizing the need for targeted interventions to support healthier dietary patterns and access to nutrition.

### 4.6. Strengths and Limitations

Our study’s primary strengths include standardized measures for assessing BMI, food consumption, and gut microbiota analysis, enhancing our findings’ reliability and comparability. However, several limitations must be acknowledged. The relatively small sample size of 91 participants may reduce the statistical power necessary to detect significant associations, especially for bacterial taxa with lower relative abundance. This limitation becomes even more pronounced when considering the complex and multifactorial relationships among diet, SES, BMI, and the diversity of gut microbiota composition. As a result, subtle but meaningful important associations between these variables and specific bacterial populations may remain undetected, limiting the study’s ability to draw robust conclusions about the intricate interplay between diet, nutritional status, social determinants, and microbial ecosystems within the human gut. Additionally, because all participants were medical students, the findings may not be generalizable to broader populations but provide relevant information on this social group. The homogeneity in education level, health behaviors, and stress factors within this group, as well as the lack of probabilistic sampling, limits the extent to which the results can be applied to non-student populations or individuals with different educational or occupational backgrounds.

A key limitation of this study is its cross-sectional design, which precludes conclusions about causality. The observed associations between gut microbiota, UPF consumption, fiber intake, and other factors cannot establish whether dietary habits directly cause changes in microbiome composition or if other variables, such as stress or genetic predispositions, are more influential. Additionally, while our analysis did not find significant associations between SES and BMI with gut microbiota composition, these factors have been shown in other research to influence microbiome diversity. Our study’s lack of significant findings may be attributed to the small sample size previously mentioned and the homogeneity of the student population, highlighting the need for future research involving more diverse populations with a broader range of SES and BMI variations. Lastly, while we considered diet and sex as variables, other important factors influencing gut microbiota, such as stress, sleep patterns, and medication use, were not thoroughly explored. Future studies should address these factors, especially in populations like medical students, who may experience high stress levels, which could confound the relationship between diet and gut microbiota composition. Additionally, the observed sex-specific differences in gut microbiota warrant further exploration of the biological mechanisms driving these differences. Research should explore the underlying mechanisms explaining why men and women have different gut microbial responses to diet and whether these differences are driven by hormonal, metabolic, or other biological factors.

## 5. Conclusions

This study sheds light on the intricate relationships between diet, SES, BMI, sex, and gut microbiota composition among medical students. Our findings suggest that consuming UPFs may negatively impact gut health, while dietary fiber intake offers protective benefits. However, the absence of significant associations with SES and BMI underscores the complexity of these interactions and suggests that additional factors may modulate these relationships.

The small sample size and the homogeneity of the study population (medical students) limit the generalizability of our findings. Therefore, caution is warranted when interpreting these results. Future research with larger and more diverse samples is crucial for our findings’ external validity and expansion. Longitudinal studies and controlled dietary interventions will also be essential to establish causal pathways between diet and gut microbiota composition.

Additionally, the sex-specific differences observed in this study highlight the need for future research to incorporate sex as a variable in the analysis, as biological and behavioral differences may influence diet–gut microbiota dynamics.

Finally, interdisciplinary efforts will be vital in advancing our understanding of how these complex factors influence gut health among students and other populations.

## Figures and Tables

**Table 1:** Characteristics of the population.

Characteristics	Total (n = 91) n (%)	Women (n = 65) n (%)	Men (n = 26) n (%)	*p*-Value *
**Age (p50, IQR)**	20 (19, 21)	20 (19, 21)	19.5 (19, 21)	0.487
**Socioeconomical status (AMAI rule)**				
Low (D+, D−)	23 (25.3)	18 (27.7)	5 (19.2)	0.399
Medium (C+, C, C−)	56 (61.5)	39 (60.0)	17 (65.4)	0.632
High (A/B)	12 (13.2)	8 (12.3)	4 (15.4)	0.693
**Body mass index (kg/m^2^) (p50, IQR) (n = 88)**	24.0 (21.3, 26.7)	24.4 (21.2, 26.7)	23.7 (22.1, 26.4)	0.763
Normal weight	53 (60.2)	38 (61.3)	15 (57.7)	0.751
Overweight + obesity	35 (39.8)	24 (38.7)	11 (42.3)	0.751
**UPF consumption**				
Above national consumption	42 (46.1)	36 (55.4)	6 (23.1)	0.005
Below national consumption	49 (53.9)	29 (44.6)	20 (76.9)	0.005
**Fiber intake**				
<25 g per day	55 (60.4)	41 (63.1)	14 (53.9)	0.417
≥25 g per day	36 (39.6)	24 (36.9)	12 (46.1)	0.417

* Differences between medians were evaluated using the two-sample Wilcoxon rank-sum (Mann–Whitney) test, and the immediate proportions test was used for the qualitative variables.

**Table 2:** The energy contribution of ultra-processed foods.

Characteristics (n = 91)	Calories from UPF Median (IQR)	Tertiles of Energy Contribution of UPF (%)			*p*-Value *
		First (n = 31)	Second (n = 30)	Third (n = 30)	
**All participants**	863 (462.1)	34.1	33	33	
Men	783.7 (368.4)	57.6	30.7	11.5	0.004
Woman	895.8 (485.6)	24.6	33.8	41.5	
**Body mass index categories**					
Normal weight	749.0 (400.8)	41.5	30.1	28.3	0.202
Overweight + obesity	916.9 (608.8)	23.6	36.8	39.4	

* Differences between medians were evaluated using the *X*^2^ test.

## Data Availability

Data are unavailable to ensure the confidentiality and protection of personal information provided by participants in our study.

## Appendix A

**Table A1:** Frequency of ultra-processed food consumption in medical students according to the NOVA classification.

Groups of Ultra-Processed Foods	Total n = 91 n (%)	Men n = 26 n (%)	Women n = 65 n (%)	*p*-Value
Industrialized drinks				
Soft drinks	32 (35.1)	7 (26.9)	25 (38.4)	0.807
Flavored milk	65 (71.4)	20 (77)	45 (69.2)	0.639
Nectars	47 (51.6)	15 (57.6)	32 (49.2)	0.572
Beverages with artificial sweeteners	68 (74.7)	21 (80.7)	47 (72.3)	0.507
Energy drinks	80 (87.9)	21 (80.7)	59 (90.7)	0.058
Fast food				
Sandwich	39 (42.8)	15 (57.6)	24 (37)	0.139
Pizza	43 (47.3)	10 (38.5)	33 (50.8)	0.461
Hamburger	58 (63.7)	16 (61.5)	42 (64.6)	0.059
Hot dog	19 (20.8)	5 (26.3)	14 (73.6)	0.874
Instant soup				
Dehydrated in a glass	70 (76.9)	20 (76.9)	50 (76.9)	1.000
Canned soup	87 (95.6)	24 (92.3)	63 (96.9)	0.428
Sweet snacks				
Candies	26 (28.5)	9 (34.6)	17 (26.1)	0.810
Chocolates	29 (31.8)	10 (38.4)	19 (29.2)	0.404
Spicy candies	49 (53.8)	15 (57.6)	34 (52.3)	0.952
Water-based ice cream	60 (65.9)	16 (61.5)	44 (67.6)	0.830
Ice creams	68 (74.7)	21 (80.7)	47 (72.3)	0.587
Bakery and biscuits				
Cookies	47 (51.6)	16 (61.5)	31 (47.9)	0.378
Hot cake	65 (71.4)	17 (65.3)	48 (73.8)	0.256
Donuts	64 (70.3)	16 (61.5)	48 (73.8)	0.278
Cakes	55 (60.4)	17 (65.3)	38 (58.4)	0.126
Bread				
Boxed bread	40 (43.9)	15 (57.6)	25 (34.8)	0.837
Industrialized cereals				
Corn tortilla	59 (64.8)	19 (73)	40 (61.5)	0.445
Boxed cereal	63 (69.2)	20 (76.9)	43 (66.1)	0.123
Cereal bars	79 (86.8)	25 (96.1)	54 (83)	0.584
Popcorn	65 (71.4)	19 (73)	46 (70.7)	1.000
Processed meat				
Ham	31 (34)	8 (30.7)	23 (35.3)	0.673
Sausages	55 (60.4)	17 (65.3)	38 (58.4)	0.812
Bacon	79 (86.8)	20 (76.9)	59 (90.7)	0.159
Chicken nuggets	16 (17.5)	6 (23)	10 (15.3)	0.455
Mortadella	90 (98)	26 (100)	64 (98.4)	0.714
Dairy drinks				
Flavored milk	65 (69.2)	20 (28.5)	45 (71.4)	0.639
Fruit yogurt	61 (45)	17 (65.3)	44 (67.6)	0.063
Drinkable yogurt	76 (83.5)	23 (88.4)	53 (81.5)	0.821
Fried foods				
Fried chicken	57 (62.6)	15 (57.6)	42 (64.6)	0.031
French fries	53 (58.2)	20 (76.9)	33 (50.7)	0.057
Dressings				
Mayonnaise	46 (70.7)	14 (53.8)	32 (49.2)	0.752
Ketchup	54 (59.3)	17 (65.3)	37 (56.9)	0.153
Worcestershire sauce	82 (90.1)	23 (88.4)	59 (90.7)	1.000
Sugar substitutes	87 (95.6)	26 (100)	61 (93.8)	0.681
Jam	65 (71.4)	18 (69.2)	47 (72.3)	0.864

* Differences between sexes were evaluated using the Fisher exact test.
